# “Sickenin’ in the rain” – increased risk of gastrointestinal and respiratory infections after urban pluvial flooding in a population-based cross-sectional study in the Netherlands

**DOI:** 10.1186/s12879-019-3984-5

**Published:** 2019-05-02

**Authors:** Annemieke Christine Mulder, Roan Pijnacker, Heleen de Man, Jan van de Kassteele, Wilfrid van Pelt, Lapo Mughini-Gras, Eelco Franz

**Affiliations:** 1 Centre for Infectious Disease Control, National Institute for Public Health and the Environment (RIVM), Antonie van Leeuwenhoeklaan 9, 3721MA, Bilthoven, Utrecht, the Netherlands; 2 Sanitas Water, Zeist, Utrecht, the Netherlands; 3 Faculty of Veterinary Medicine, Utrecht University, Utrecht, the Netherlands

**Keywords:** urban flooding, floodwater exposure, climate change, AGE, ARI

## Abstract

**Background:**

Climate change is expected to increase the chance of extreme rainfall events in the Northern Hemisphere and herewith, there is an increased chance of urban pluvial flooding. Urban pluvial flooding often consists of street flooding and/or flooding of combined sewerage systems, leading to contamination of the floodwater with several gastrointestinal and/or respiratory pathogens. An increase in flooding events therefore pose a health risk to those exposed to urban floodwater. We studied the association between exposure to pluvial floodwater and acute gastroenteritis (AGE) and acute respiratory infection (ARI).

**Methods:**

We performed a retrospective, cross-sectional survey during the summer of 2015 in 60 locations in the Netherlands with reported flooding. Two weeks after the flooding, questionnaires were sent to households in these locations, collecting data on self-reported AGE and ARI and information on floodwater exposure in the previous 2 weeks. Multivariable generalized estimating equations (GEE) regression models, accounting for the clustered data structure, were used to identify risk factors for AGE and ARI.

**Results:**

In total, 699 households with 1,656 participants (response rate 21%) returned the questionnaire. Contact with floodwater was significantly associated with AGE (aOR 4.2, 95%CI 2.1–8.4) and ARI (aOR 3.3, 95%CI 2.0–5.4). Risk factors for AGE were skin contact with floodwater (aOR 4.0, 95%CI 1.8-9.0), performing post-flooding cleaning operations (aOR 8.6, 95%CI 3.5-20.9) and cycling through floodwater (aOR 2.3, 95%CI 1.0-5.0). Skin contact with floodwater (aOR 3.6, 95%CI 1.9-6.9) and performing post-flooding cleaning operations (aOR 5.5, 95%CI 3.0-10.3) were identified as risk factors for ARI.

**Conclusions:**

Results suggest an association between direct exposure to pluvial floodwater and AGE and ARI. As it is predicted that the frequency of pluvial flooding events will increase in the future, there is a need for flood-proof solutions in urban development and increased awareness among stakeholders and the public about the potential health risks. Future prospective studies are recommended to confirm our results.

**Electronic supplementary material:**

The online version of this article (10.1186/s12879-019-3984-5) contains supplementary material, which is available to authorized users.

## Background

Climate change leads to extreme weather events that can have devastating effects on human society and the environment [1]. Climate change models predict an almost exponential increase in atmospheric water-holding capacity, with increasing temperature and subsequent rise in atmospheric water content leading to an increase in extreme rainfall events in the Northern Hemisphere [2]. As extreme rainfall events are expected to increase in frequency, intensity and duration, pluvial flooding events are also expected to increase in urban settings [3]. Indeed, in the last few decades, countries like the Netherlands have observed an increasing trend in extreme rainfall events, causing recurrent pluvial flooding, especially in urban areas [4].

Most urban sewage drainage systems in the Netherlands can only support an intensity of ~20 mm of rainfall per hour. Extreme rainfall events (usually > 30 mm rainfall/hour for > 1 h) may overwhelm this drainage capacity, leading to 10-50 cm of pluvial floodwater to accumulate on the surface [3, 5]. Urban pluvial flooding often entails street flooding and/or flooding of combined sewerage system, where rainwater mixes with sewage water, thereby heavily contaminating floodwater with fecal material. In this way, floodwater becomes a possible vehicle of several pathogens such as noroviruses, enteroviruses, or *Campylobacter* [6], many of which are recognized causative agents of gastrointestinal and respiratory infections.

People will inevitably be exposed to this floodwater, especially in urban settings and when engaged in post-flooding cleaning or recreational activities (e.g. swimming, playing in water, etc.). It is also possible that people are accidentally exposed to floodwater when passing through it (e.g. walking, cycling, etc.) or because of splash exposure. Increased flooding events may therefore pose a threat for public health, particularly for the development of acute gastroenteritis (AGE) or acute respiratory infection (ARI) via ingestion and inhalation of, and dermal contact with, pathogens in floodwater as is shown by the study of De Man, et al. [6]. However, little research is available on health risks associated with urban pluvial flooding.

With a focus on a high-income country like the Netherlands, the aim of this study was to quantify the AGE and ARI risks associated with exposure to pluvial floodwater, as well as to identify specific risk factors for AGE and ARI, in pluvial flood-ravaged urban areas.

## Methods

### Study design

To collect data on self-reported AGE and ARI after urban pluvial flooding, a retrospective cross-sectional survey was conducted during the summer of 2015 using the methodology described in De Man, et al. [3]. In total, 3382 households that were surrounded by floodwater after extreme rainfall events (extreme rainfall events are rainfall events for which streets are flooded) in 60 locations in the Netherlands received a self-administered questionnaire by regular post. The questionnaire was send 2 weeks after the start of the flooding event and participants were asked to answer questions considering a recall period of 2 weeks, i.e. from the start of the flooding until reception of the questionnaire. Flood locations were identified by monitoring possible flood events on the internet, such as social media and press releases, which generally receive much media attention in the Netherlands. The national website of the Dutch fire brigade was the main source of information to determine which households were surrounded by floodwater (http://www.112meldingen.nl/). This website provides the causes of a given alert, which the Dutch fire brigade receives from the citizens themselves, corroborates on-site, and disseminates to the public, specifying the affected areas (postal codes). With this information, the addresses of the affected areas to which the questionnaires were sent were derived from publicly available mapping tools like googlemaps.com [3].

### Data collection

The questionnaire was developed by adapting the questionnaire used in the study of De Man, et al. [3] to collect epidemiologically relevant information for each household member (individual participant). Information was collected on basic demographic characteristics (i.e. age, sex, number of household members). Furthermore, information was gathered about the occurrence of gastrointestinal and respiratory complaints during the 2 weeks before questionnaire completion, underlying chronic diseases, type of exposure (i.e. contact) to floodwater, and type of activity leading to contact with floodwater, duration of flooding in minutes and the magnitude of exposure (height of floodwater in cm). Participants reporting one or more types of contact or types of activities leading to contact with floodwater are hereafter referred to as being exposed to floodwater.

Questions could be answered for up to five household members (most Dutch households are composed of ≤ 4 people according to Netherlands Statistics, https://www.cbs.nl/, [7]), and the participant was asked to report information on all household members.

### Ethical considerations

This study involved collection and analysis of fully anonymized data, so no ethical approval was necessary according to Dutch regulations. [8, 9] People gave consent upon anonymously completing and returning the questionnaire. Questionnaires were received in de-identified form, containing only data on postal code, sex, and age of the participants. Therefore, names and addresses could not be linked to the questionnaire responses, guaranteeing anonymity of the respondents. Participants were informed that the data they provided were to be analyzed for scientific purposes, and that by returning the questionnaires, they gave consent to do so. No written consent was therefore necessary. Parents were asked to complete the questionnaire and give consent on behalf of their children (family members < 16 years old).

### Case definitions

Based on the symptoms reported in the questionnaire, participants were defined as cases of AGE or ARI when experiencing the following complaints in the 2 weeks before completing the questionnaire, and as non-cases otherwise:

- AGE: any individual experiencing ≥ 3 diarrheal discharges per day or any clinically relevant vomiting (i.e. vomiting events other than regurgitation, vomiting due to motion sickness/vertigo, traumatic event, nauseous event, or drug/alcohol abuse) [10].
- ARI: acute onset of symptoms (within 2 weeks before completion of the questionnaire) and common cold, shortness of breath, coughing, sore throat, wheezing, chest pain or sneezing [11].

### Data analysis

Generalized estimating equations (GEEs) were used to compare AGE and ARI in cases with the non-cases, with regard to their type of contact and type of activity leading to contact with floodwater. As our study design led to clustering of data at the household-level, we corrected for dependence of observations deriving from individuals living in the same household [12]. An exchangeable working correlation structure was chosen for the GEE models, meaning that the correlation between individuals within a cluster was the same for all clusters. An overall model was built for all age categories (categorical: 0-5, 6-15, 16-25, 26-45, 46-65 and > 65 years), as well as separate models for adults (categorical: 16-25, 26-45, 46-65 and > 65 years) and for children (< 16 years, continuous), which were further subdivided in a model for type of exposure and a model for type of activity leading to exposure.

Variables with a *p*-value ≤ 0.20 in the univariate analyses were selected for inclusion in multivariable GEE logistic regression models. Potential confounders that were always adjusted for in multivariable analyses were age category, sex (male, female, missing) and summer (summer: June, July, August; not-summer: other months; missing: NAs). Additionally, models for AGE were corrected for chronic disorders: disease of the gastrointestinal tract (yes/no), reflux (yes/no), food allergy (yes/no), or pregnancy with vomiting during the study period. Models for ARI were corrected for hay fever (yes/no), and lung anomalies (e.g. asthma, chronic obstructive pulmonary disease – COPD, etc.; yes/no).

A manual backward-forward selection procedure was used to retain only those variables with p ≤ 0.05. Collinear variables were selected based on improved model fit as revealed by the Quasi-likelihood under the Independence model Criterion (QIC) statistic [13]. Biologically plausible interactions between independent, correlated variables were also assessed. Interactions were first tested in the univariate analyses (only interaction terms were included, confounders and other possible risk factors were added later in the multivariate analyses), and those with a *p*-value ≤ 0.20 were included in the multivariable model. We present the adjusted odds ratios (aOR) and 95% confidence intervals from the final multivariable models. For results from the univariate analyses, see Additional file 1: Table S1 and Additional file 2: Table S2.

We used R (version 3.4.3) [14] and the gee package (version 4.8) [15] for data analyses.

## Results

### Sample description

**Fig. 1:**
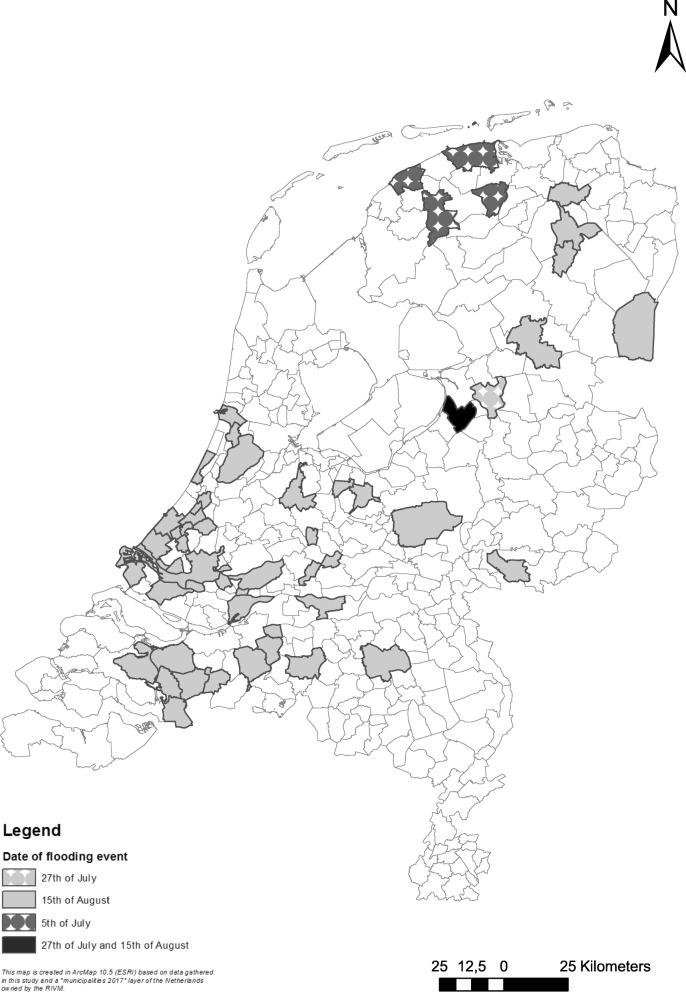
Map showing the municipalities that were affected by pluvial flooding at 05-07-2015, 27-07-2015 and 15-08-2017

In total, 3382 households were invited and 699 households (response 21%; 1,656 individual participants) completed the questionnaire after three extreme rainfall events caused urban pluvial flooding on respectively July 5th, July 27th and August 15th, 2015, in 60 municipalities in the Netherlands (Fig. 1). On average, the participants returned the questionnaire 9 days after sending (median = 6 days).

**Fig. 2:**
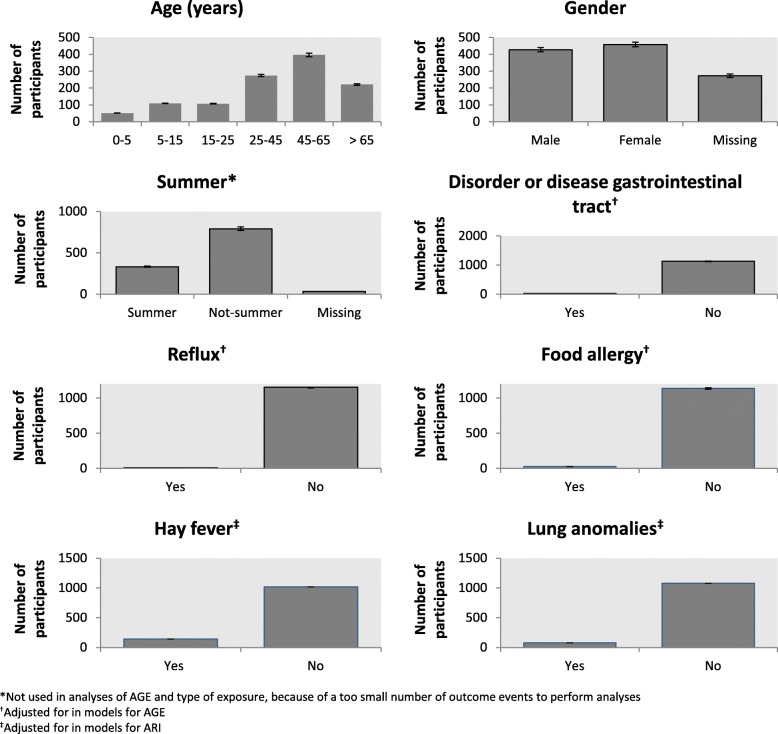
General characteristics of the study participants

Twenty-one individual participants (1.3%) were excluded, because information reported on exposure was contradictory. Information about complaints and exposure was incomplete for 477 individual participants (28.8%). Therefore, they were also eliminated from the analysis (see Additional file 3: Figure S1). The remaining 1,158 individual participants (582 households) represented the study population of which we had information for both cases and non-cases (Fig. 2). There were no pregnant participants, who also reported vomiting during the recall period (see Additional file 3: Figure S1). Table 1 gives an overview of the descriptive statistics. 277 participants (24%) have an underlying chronic disease, 690 participants (60%) were exposed to floodwater (i.e. contact) and 966 participants (83%) performed a type of activity leading to contact with floodwater.

**Table 1:** Descriptive statistics of the risk factors of developing health complaints after contact with floodwater

Risk factors	Total number of participants N (%)	Total number of adults N (%)	Total number of children N (%)
Chronic diseases			
Chronic disorder or disease of the gastrointestinal tract	27 (2)	27 (3)	0 (0)
Reflux	8 (1)	7 (1)	1 (1)
Food allergy	23 (2)	18 (2)	5 (3)
Pregnancy with vomiting	0 (0)	0 (0)	0 (0)
Hay fever	140 (12)	124 (12)	16 (9)
Lung anomalies	79 (7)	68 (7)	11 (6)
No chronic diseases	881 (76)	738 (75)	143 (81)
Type of exposure			
Skin contact	527 (46)	457 (47)	70 (40)
Droplets of floodwater in the mouth	19 (2)	15 (2)	4 (2)
Gulp of floodwater in the mouth	139 (12)	113 (11)	26 (15)
Head submerged in floodwater	5 (0)	4 (0)	1 (0)
Not exposed	468 (40)	393 (40)	75 (43)
Type of activity			
Cleaning inside	320 (28)	311 (32)	9 (5)
Cleaning outside	213 (18)	208 (21)	5 (3)
Swum in floodwater	3 (0)	0 (0)	3 (2)
Used rubber boat	7 (1)	4 (0)	3 (2)
Walked through floodwater	198 (17)	158 (16)	40 (23)
Cycled through floodwater	86 (7)	71 (7)	15 (8)
Driven through floodwater by car	139 (12)	135 (14)	4 (2)
No activities	192 (17)	95 (10)	97 (55)
Location of flooding			
Toilet overflow	250 (22)	209 (21)	41 (23)
No toilet overflow	908 (78)	773 (79)	135 (77)
Street flooding	779 (67)	650 (66)	129 (73)
No street flooding	379 (33)	332 (34)	47 (27)

**Fig. 3:**
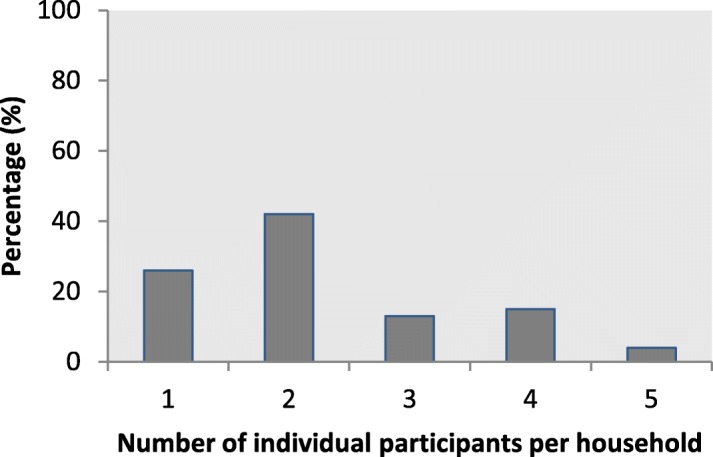
Percentage of participating households with 1, 2, 3, 4 or 5 individual participants

In the study population, 40% were women, 37% were male, and of 23% of the participants the sex was unknown. The median age was 47 years (25-75% percentile P_25-75_ 27 – 61). Compared to the Dutch general population in 2015 (16,900,726 inhabitants [16]), the sample contained slightly more women (52% vs. 51%) and was slightly older (average age 43 *vs.* 41 years). Most households (281;42%) reported two individual participants and 28 households (4%) reported five individual participants (Fig. 3).

### Prevalence of AGE and ARI

During the 2-week period after flooding, 75 (12.3%) participants in 51 households reported AGE, and 128 (21.1%) participants in 114 households reported ARI (Table 2), after being exposed. Table 2 also shows the results of the univariate and multivariate analyses. In the multivariate analyses, exposure to floodwater was significantly associated with increased odds of AGE (aOR 4.2, 95% CI 2.1 – 8.4). Furthermore, exposure to floodwater was associated with increased odds of reporting ARI (aOR 3.3, 95% CI 2.0 – 5.4). The analyses on the association between floodwater exposure and AGE in children < 16 years could not be performed given the small number of outcome events. Indeed, there were no children without contact with floodwater that also had AGE. Furthermore, Table 2 shows that there was no significant association between floodwater exposure and ARI in children (OR 1.6, 95% CI 0.6 – 4.0) in the univariate analysis. In adults, floodwater exposure was associated with both AGE and ARI (aOR 3.6, 95% CI 1.8 – 7.3, and aOR 4.0, 95% CI 2.3 – 6.8, respectively).

**Table 2:** Health complaints of individual participants and the association with exposure to pluvial floodwater

	Without exposure *N* = 550	With exposure *N* = 608	OR	aOR	Children without exposure *N* = 95	Children with exposure *N* = 81	OR	aOR	Adults without exposure *N* = 455	Adults with exposure *N* = 527	OR	aOR
	N (%)	N (%)	(95% CI)	(95% CI)	N (%)	N (%)	(95% CI)	(95% CI)	N (%)	N (%)	(95% CI)	(95% CI)
AGE	12 (2)	75 (12)	**4.9 (2.5, 9.6)**	**4.2** ^**a**^ **(2.1, 8.4)**	0 (0)	12 (15)	-	-	12 (3)	63 (12)	**4.2 (2.2, 8.2)**	**3.6** ^**a**^ **(1.8, 7.3)**
ARI	38 (7)	128 (21)	**3.5 (2.2, 5.7)**	**3.3** ^**b**^ **(2.0, 5.4)**	10 (11)	14 (17)	1.6 (0.6, 4.0)	-	28 (6)	114 (22)	**4.1 (2.5, 6.7)**	**4.0** ^**b**^ **(2.3, 6.8)**

Note: There were no children without contact with the floodwater, but with AGE, leading to a too small number of outcome events to perform the univariate analysis. Furthermore, there were not enough children to prevent the same from happening in the multivariate analyses for contact with the floodwater and ARI

^a^Adjusted for age, sex, summer, having a chronic disorder or disease of the gastrointestinal tract, having reflux, having a food allergy or pregnancy with vomiting during the study period

^b^Adjusted for age, sex, summer, having hay fever and having lung anomalies (e.g. asthma, chronic obstructive pulmonary disease - COPD, etc.)

Factors with a p-value <0.05 are expressed in bold

### Multivariate analyses

Results from the final multivariable models for AGE and ARI are presented in Tables 3 and 4 respectively. For results from the univariate models, see Additional file 1: Table S1 and Additional file 2: Table S2.

**Table 3:** Results of multivariate analyses for AGE in all age categories (overall), children (<16years) and adults

Model & Covariates	Overall without AGE	Overall with AGE	aOR	Children without AGE	Children with AGE	aOR	Adults without AGE	Adults with AGE	aOR
	N (%)	N (%)	(95% CI) ^a^	N (%)	N (%)	(95% CI) ^a^	N (%)	N (%)	(95% CI) ^a^
Type of exposure									
Skin contact	456 (70)	71 (93)	**4.0 (1.8, 9.0)**	59 (65)	11 (100)	NA	397 (71)	60 (92)	**3.8 (1.5, 9.4)**
Type of activity									
Water contact									
No (ref)			ref			ref			ref
Yes, no cleaning	190 (18)	22 (25)	**2.9 (1.3, 6.7)**	NA	NA	NA	128 (14)	12 (16)	1.5 (0.5, 4.3)
Yes, cleaning inside	173 (16)	17 (20)	**3.7 (1.4, 9.6)**	NA	NA	NA	167 (18)	17 (23)	**3.3 (1.3, 8.4)**
Yes, cleaning outside	69 (6)	14 (16)	**6.9 (2.5, 18.7)**	NA	NA	NA	69 (8)	12 (16)	**5.3 (1.9, 14.7)**
Yes, cleaning in & out	106 (10)	24 (28)	**8.6 (3.5, 20.9)**	NA	NA	NA	103 (11)	24 (32)	**7.6 (3.1, 18.6)**
Cycled	62 (6)	24 (28)	**2.3 (1.0, 5.0)**	10 (6)	5 (42)	NA	52 (6)	19 (25)	**2.4 (1.0, 5.7)**
Do not know	5 (0)	2 (2)	NA	2 (1)	0 (0)	NA	3 (0)	2 (3)	**27 (4.7, 158)**

Note*: aOR* adjusted odds ratio, *CI* confidence interval, *NA* not applicable, overall analyses of exposure vs AGE could not be performed for children, *ref* reference category

^a^Adjusted for age, sex, summer, having a chronic disorder or disease of the gastrointestinal tract, having reflux, having a food allergy or pregnancy with vomiting during the study period

Factors with a *p*-value <0.05 are expressed in bold

**Table 4:** Results of multivariate analyses for ARI in all age categories (overall), children (<16years) and adults

Model & Covariates	Overall without ARI	Overall with ARI	aOR	Children without ARI	Children with ARI	aOR	Adults without ARI	Adults with ARI	aOR
	N (%)	N (%)	(95% CI) ^a^	N (%)	N (%)	(95% CI) ^a^	N (%)	N (%)	(95% CI) ^a^
Type of exposure									
Skin contact	406 (68)	121 (88)	**3.6 (1.9, 6.9)**	54 (64)	16 (89)	NA	352 (69)	105 (88)	**3.7 (1.8, 7.7)**
Type of activity									
Water contact									
No (ref)			ref			ref			ref
Yes, no cleaning	168 (17)	44 (27)	**3.0 (1.6, 5.7)**	NA	NA	NA	112 (13)	28 (20)	**3.8 (1.9, 7.6)**
Yes, cleaning inside	155 (16)	35 (21)	**4.0 (2.2, 7.4)**	NA	NA	NA	149 (18)	35 (25)	**4.3 (2.2, 8.4)**
Yes, cleaning outside	61 (6)	22 (13)	**4.4 (2.2, 8.7)**	NA	NA	NA	59 (7)	22 (15)	**5.2 (2.5, 10.7)**
Yes, cleaning in & out	98 (10)	32 (19)	**5.5 (3.0, 10.3)**	NA	NA	NA	95 (11)	32 (23)	**5.7 (2.9, 11.3)**
Cycled	61 (6)	25 (15)	NA	12 (8)	3 (13)	NA	49 (6)	22 (15)	NA
Do not know	2 (0)	5 (3)	**9.4 (1.4, 64.9)**	0 (0)	2 (8)	NA	2 (0)	3 (2)	NA

Note: *aOR* adjusted odds ratio, *CI* confidence interval, *NA* not applicable (overall analyses of exposure vs AGE could not be performed for children), *ref* reference category

^a^Adjusted for age, sex, summer, having hay fever and having lung anomalies (e.g. asthma, chronic obstructive pulmonary disease - COPD, etc.)

Factors with a *p*-value <0.05 are expressed in bold

In the models for type of activity and AGE or ARI, an interaction term was included between contact with floodwater and cleaning floodwater inside or outside the house. The strata included in this interaction term are: no contact with floodwater at all (reference category), contact with floodwater but no involvement in post-flooding cleaning operations, contact with floodwater and involvement in indoor cleaning operations, contact with floodwater and involvement in outdoor cleaning operations, and contact with floodwater and involvement in both indoor and outdoor cleaning operations. The rationale is that people with the least exposure to floodwater have the lowest chance to get AGE or ARI (water contact without cleaning), while people with the largest exposure to floodwater (water contact with cleaning inside and outside the house) have the highest chance to get AGE or ARI.

### AGE

#### All age category

In the multivariate model assessing AGE risk in relation to the type of exposure to floodwater for all age category (overall model), having had skin contact with floodwater was significantly associated with AGE (aOR 4.0, 95% CI 1.8 – 9.0; Table 3). Regarding the type of activity, the aORs in all strata of the interaction term that included contact with floodwater were significantly increased. Indeed, the model showed increasing aORs from having had contact with floodwater without having been engaged in any cleaning operation (aOR 2.9, 95% CI 1.3 – 6.7) to having had contact with floodwater and having been engaged in cleaning operations both inside and outside the household (aOR 8.6, 95% CI 3.5 – 20.9). Cycling in floodwater was also a significant risk factor for AGE (aOR 2.3, 95% CI 1.0 – 5.0).

#### Adults

In the multivariate model assessing AGE risk in relation to the type of exposure to floodwater among adults, having had skin contact with floodwater was significantly associated with AGE (aOR 3.8, 95% CI 1.5 – 9.4; Table 3). Regarding the type of activity, the aORs in the strata of the interaction term that included contact with floodwater and cleaning were significantly increased. Indeed, the model showed increasing aORs from having had contact with floodwater and having been engaged in indoor cleaning operations (aOR 3.3, 95% CI 1.3 – 8.4) to having had contact with floodwater and having been engaged in both indoor and outdoor cleaning operations (aOR 7.6, 95% CI 3.1 – 18.6). Cycling in floodwater was also a risk factor for AGE (aOR 2.4, 95% CI 1.0 – 5.7).

#### Children

No multivariate model for AGE in children could be built because there were not enough children with AGE to perform the analysis.

### ARI

#### All age category

In the multivariate model assessing ARI risk in relation to the type of exposure to floodwater for all age category (overall model), having had skin contact with floodwater was significantly associated with ARI (aOR 3.6, 95% CI 1.9 – 6.9; Table 4). Regarding the type of activity, the aORs in all strata of the interaction term that included contact with floodwater were significantly increased. Indeed, the model showed increasing aORs from having had contact with floodwater without having been engaged in any cleaning operation (aOR 3.0, 95% CI 1.6 – 5.7) to having had contact with floodwater and having been engaged in both indoor and outdoor cleaning operations (aOR 5.5, 95% CI 3.0 – 10.3).

#### Adults

In the multivariate model assessing ARI risk in relation to the type of exposure to floodwater among adults, having had skin contact with floodwater was significantly associated with ARI (aOR 3.7, 95% CI 1.8 – 7.7; Table 4). Regarding the type of activity, the aORs in all strata of the interaction term that included contact with floodwater were significantly increased, with increasing aORs from having had contact with floodwater but no involvement in cleaning operations (aOR 3.8, 95% CI 1.9 – 7.6) to having had contact with floodwater and having been involved in cleaning operations both inside and outside the household (aOR 5.7, 95% CI 2.9 – 11.3).

#### Children

There was no significant association between exposure to floodwater and ARI for children.

### Magnitude of exposure

The average height of the water (magnitude of exposure) in the streets was 19 cm (median = 15 cm, height _min_ = 2 cm, height _max_ = 150 cm). The average height of the water after toilet overflow was 5 cm (median = 2 cm, height _min_ = 1 cm, height _max_ = 30 cm). Additional univariate analyses showed that the magnitude of exposure in the streets was a risk factor for both AGE (OR 1.0; 95% CI 1.0 – 1.1) and ARI (toilet overflow, OR 1.1; 95% CI 1.0 – 1.2).

## Discussion

This study suggests an association between direct exposure to urban pluvial floodwater and the occurrence of both AGE and ARI in a high-income country like the Netherlands. Identified risk factors were contact with floodwater, such as skin contact with floodwater (for both AGE and ARI), post-flooding cleaning operations (for both AGE and ARI) and cycling through floodwater (for AGE).

This study included 1656 individual participants, resulting in a response rate of 21%. It is unknown how representative our sample is with regard to the target population (i.e. the people who experienced flooding), as we did not know the demographics of the people invited to participate in the study. Addressee-unknown invitations were sent to all house numbers of streets in which flooding had been reported. Therefore, it was impossible for us to know who the invitees were. It could be argued that representativeness of the sample could be assessed based on the demographics of the Dutch general population. However, this is not an optimal solution, because the ‘target population’, i.e. the people who experienced flooding, does not necessarily mirror the general population.

A response rate of 20-30% is commonly reported in this type of retrospective studies where self-reported health complaints are investigated [17, 18], and some studies report even lower response rates [19]. A low response rate could render these studies prone to, for example, selection bias [18]. The type of selection bias that might have played a role in this study is self-selection bias, by which the group of participants who were exposed and diseased is overrepresented, as people who experienced flooding and health complaints are particularly motivated to complete and return the questionnaire [18]. Another limitation that is inherent to retrospective studies that use self-reported data is recall bias, where people might have forgotten (mild) AGE and ARI episodes [18]. On the other hand, an overestimation of the incidence might also have occurred, because of ‘telescoping’ (i.e. when people remember episodes as being more recent than they actually are) [18]. Overall, retrospective studies with self-reported data like ours tend to produce incidence estimates that overestimate the true incidence [20, 21]. A major advantage of these type of studies is that they allow for collection of data about ARI and AGE cases that are not reported to the General Practitioner (GP), i.e. cases that do not require medical attention.

Previous population-based studies on AGE in the Netherlands showed a baseline incidence of 0.95 episodes/person-year. In our study targeting pluvial flood-ravaged areas, the incidence of AGE was estimated at 1.69 episodes/person-year, which is almost twice as higher. This could suggest that flooding events increase AGE risk in the affected population also as compared to the baseline AGE incidence in the whole country [19]. Likewise, the incidence of ARI derived from our study was 3.74 episodes/person-year, which is more than twice as higher than the incidence of influenza-like illness (ILI) in the general Dutch population (1.72 episodes/person-year) [18]. Although ARI is not the same as ILI, they both include respiratory diseases and give an indication of the number of cases with respiratory disease in the country. However, direct comparison with the ARI/AGE incidence of the general population is hampered by the fact that we used a shorter recall period (2 vs. 4 weeks), which generally produces higher incidence estimates [20, 21]. Moreover, our study might have been subject to reporting and selection bias, as is described above.

De Man, et al. [3] performed a comparable study in the Netherlands at a much smaller scale (149 households) and lacked sufficient numbers of outcome events to use well-defined (standardized) AGE or ARI syndrome definitions. The larger scale of our study (699 households) allowed for estimates that are more precise and for the use of standardized definitions for both AGE and ARI. In agreement with De Man, et al. [3], we found that participants exposed to pluvial floodwater were more likely to develop gastrointestinal and respiratory complaints (Table 2).

We also investigated differences in risk factors for AGE and ARI in children and adults, but the number of children enrolled in the study was rather low, so the analyses for this age category were underpowered (Table 2). This may be reason as to why the association between ARI and exposure to floodwater was positive but not statistically significant. Low statistical power did also not allow De Man, et al. [3] to study differences in risk factors for AGE and ARI between adults and children. However, it is evident that children may display certain risk factors (e.g. playing in/with floodwater) more often than adults (e.g. post-cleaning operations). It was also shown by De Man, et al. [6] that children are more likely to ingest floodwater compared to adults (1.7 ml vs. 0.016 ml), because they play in or around floodwater, and therefore have a higher risk of AGE (33% vs. 3.9%).

Sanitary sewer overflow (SSOs) events were shown to be associated with gastrointestinal illness (GI; emergency room (ER) visits with a primary diagnosis of GI) [22]. However, specific risk factors leading to exposure and eventually GI were not identified. Furthermore, SSOs probably entail different risk factors (swimming, contaminated drinking water) compared to flooding of combined sewerage systems/street flooding as studied in this paper.

For ARI and AGE, skin contact was identified as a risk factor, which is probably a proxy for ingestion or inhalation of contaminated floodwater (Tables 3 and 4). This may happen, for example, when people do not wash their hands after floodwater contact or people splash aerosolized water particles in their face, while only reporting skin contact [23]. This could possible also explain why for example ingesting droplets of floodwater was not identified as risk factor, as people only report the most evident risk factors, i.e. skin contact.

Post-flooding cleaning operations were associated with increased risk for both ARI and AGE (Tables 3 and 4), which was only associated with ARI in De Man, et al. [3]. It could be that we were able to identify it as a risk factor for AGE due to our larger sample size. Our finding is supported by previous literature describing that cleaning leads to aerosolisation of contaminated water droplets and their inhalation/ingestion, explaining why it could be a risk factor for AGE [23]. Similarly, cycling through floodwater was a significant exposure for AGE (Table 3), as water droplets could splash into their face and mouth. As reported in De Man, et al. [3], these associations may reflect, to some extent, the primary transmission routes of the pathogens in question, i.e. inhalation for ARI and ingestion for AGE. Indeed, several are typically associated with flood-ravaged settings in developed countries. Sales-Ortells and Medema [24] found, for example, *Campylobacter* in all water plaza samples in which people recreate, leading to a risk of developing AGE for those people.

A limitation of this study is that there were no water samples obtained from pluvial floodwater as well as no faecal samples from the participants. Therefore, the causative agents remain unknown. However, pluvial floodwater in the Netherlands was shown to always be contaminated with faeces, as was demonstrated by the presence of *E. coli*, intestinal enterococci, and enteropathogens such as enterovirus, norovirus and *Campylobacter* in water samples of pluvial floodwater [6].

Furthermore, there was no data on sex for 23% of the participants, primarily children, which was because the questionnaire only collected information regarding sex of the person filling in the questionnaire, but not for other household members. This explains most of the missings and is unlikely to affect our results because sex effects on AGE and ARI are less likely to be seen in children [19, 25]. Moreover, we do not know whether participants used a measure tape to measure the height of the floodwater in cm to answer the question. Although the magnitude of exposure came out as a risk factor in the analysis for AGE and ARI, those data could potentially be erroneous as most of the collected data are self-reported estimates of the true height of the floodwater.

Chronic diseases, which the models were corrected for, may be effect modifiers of the association between floodwater exposure and AGE/ARI. This was checked by running the models with and without chronically diseased cases therein: results can be found in Additional file 4: Table S3. They show that chronic diseases were not significant effect modifiers. However, they were associated with the outcome, so the models were always corrected for underlying chronic disease.

Future prospective studies are needed to confirm the associations found in this study. An example of such a study could be a longitudinal study that monitors the rainfall pattern in a given area. As soon as an extreme rainfall event occurs (> 30 mm rainfall/hour for > 1 h), it should be checked whether flooding has also occurred in that specific area. A representative selection of the inhabitants of the flooded area (which have been actively enrolled at the beginning of the study, perhaps upon financial incentive) should use health diaries to record their symptoms and their exposure to floodwater. This would reduce both recall and selection bias. In order to further confirm causality, floodwater and patient faeces samples should also be taken to identify and characterize potential pathogens.

Due to climate change, extreme rainfall events will occur more frequently, leading to more people being exposed to pluvial floodwater in urban areas in countries like the Netherlands [2]. As this study suggests, such events are likely to lead to increased risk of health effects. Generally, people in developed countries tend to not perceive the associated health risks. The Netherlands is a highly populated country (~400 people/km^2^) [3], so urban flooding will not pass easily unnoticed. Recently, awareness has been arisen, with Dutch residents demanding municipal services to improve drainage systems as to prevent pluvial flooding [26]. In response, governmental authorities are promoting greenness in urban areas to facilitate natural drainage of the water in the soil [26]. This study adds another perspective to this debate and shows that it is necessary to take proper care of water drainage systems/sewage systems in terms of their drainage capacity to mitigate health risks in urban areas.

## Conclusion

In conclusion, this study suggests an association between different non-mutually exclusive types of direct exposure and activities leading to exposure to floodwater and AGE and ARI, which is a possible reflection of the transmission routes of the pathogens in question. However, future prospective studies are needed to confirm this association. Since pluvial flooding events will increase in the future, proving the causal link would reinforce the need for flood-proof solutions in urban development and increased awareness among stakeholders and the public about the associated health risks.

## Additional files

Additional file 1: **Table S1.** Results of univariate analyses for AGE in all age categories (overall), children (<16years) and adults. (DOCX 24 kb)

Additional file 2: **Table S2.** Results of univariate analyses for ARI in all age categories (overall), children (<16years) and adults. (DOCX 22 kb)

Additional file 3: **Figure S1.** Flowchart discarded information. (DOCX 47 kb)

Additional file 4: **Table S3.** Additional analysis of AGE and ARI with regard to their risk factors with and without individuals having chronic diseases. (DOCX 18 kb)

## Abbreviations

AGE: Acute gastroenteritis
ARI: Acute respiratory infection
