# Supplementary material for: “Sickenin’ in the rain” – increased risk of gastrointestinal and respiratory infections after urban pluvial flooding in a population-based cross-sectional study in the Netherlands
Source: BMC Infect Dis. 2019 May 2;19:377. doi: 10.1186/s12879-019-3984-5 (PMC6498475; doi:10.1186/s12879-019-3984-5)
Supplement: Supplementary file 1 — Table S1. Results of univariate analyses for AGE in all age categories (overall), children (<16years) and adults. (DOCX 24 kb) [file 12879_2019_3984_MOESM1_ESM.docx]

Table S1: *Results of univariate analyses for AGE in all age categories (overall), children (<16years) and adults (factors with a p-value <0.20 are expressed in bold)*

| Model & Covariates | Overall  without AGE | Overall  with  AGE | OR | Children without AGE | Children  with  AGE | OR | Adults without AGE | Adults with  AGE | OR |
| --- | --- | --- | --- | --- | --- | --- | --- | --- | --- |
|  | N (%) | N (%) | (95% CI) | N (%) | N (%) | (95% CI) | N (%) | N (%) | (95% CI) |
| Type of exposure |  |  |  |  |  |  |  |  |  |
| Skin contact | 456 (70) | 71 (93) | 5.5 (2.1, 14.8) | 59 (65) | 11 (100) | NA | 397 (71) | 60 (92) | 4.9 (1.7, 13.7) |
| Droplets of water in the mouth | 16 (2) | 3 (4) | 2.4 (0.8, 7.0) | 4 (4) | 0 (0) | NA | 12 (2) | 3 (5) | 2.5 (0.8, 8.1) |
| Gulp of water in the mouth | 119 (18) | 20 (26) | 2.0 (1.1, 3.9) | 25 (27) | 1 (8) | NA | 94 (17) | 19 (29) | 1.9 (1.0, 3.5) |
| Head submerged | 4 (1) | 1 (1) | 3.3 (0.5, 23.4) | 0 (0) | 1 (8) | NA | 4 (1) | 0 (0) | NA |
|  |  |  |  |  |  |  |  |  |  |
| Type of activity |  |  |  |  |  |  |  |  |  |
| Cleaning inside | 279 (26) | 41 (47) | 2.3 (1.2, 4.4) | 9 (5) | 0 (0) | NA | 270 (30) | 41 (55) | 2.7 (1.5, 4.9) |
| Cleaning outside | 175 (16) | 38 (44) | 3.4 (2.0, 5.8) | 3 (2) | 2 (17) | NA | 172 (19) | 36 (48) | 3.7 (2.2, 6.3) |
| Played/run/splashed | 72 (7) | 17 (20) | 1.5 (0.7, 3.4) | 46 (28) | 10 (83) | NA | 26 (3) | 7 (9) | 2.4 (0.6, 8.8) |
| Swum | 2 (0) | 1 (1) | 1.3 (0.2, 9.8) | 2 (1) | 1 (8) | NA | 0 (0) | 0 (0) | NA |
| Used rubber boat | 5 (0) | 2 (2) | 2.8 (0.8, 9.5) | 2 (1) | 1 (8) | NA | 3 (0) | 1 (1) | 3.0 (0.8, 11.7) |
| Walked | 165 (15) | 33 (38) | 2.8 (1.6, 4.7) | 35 (21) | 5 (42) | NA | 130 (14) | 28 (37) | 3.0 (1.7, 5.3) |
| Cycled | 62 (6) | 24 (28) | 4.5 (2.2, 8.9) | 10 (6) | 5 (42) | NA | 52 (6) | 19 (25) | 4.7 (2.3, 9.4) |
| Driven | 117 (11) | 22 (25) | 1.5 (0.9, 2.7) | 4 (2) | 0 (0) | NA | 113 (12) | 22 (29) | 1.8 (1.0, 3.4) |
| Do not know | 5 (0) | 2 (2) | 1.6 (0.0, 230) | 2 (1) | 0 (0) | NA | 3 (0) | 2 (3) | 7.7 (1.4, 43.1) |

Note: OR, odds ratio; CI, confidence interval; NA, not applicable (overall analyses exposure vs AGE was not applicable for children, so the separate univariate analyses, multivariate model and the interaction term for children were not created)
