# Supplementary material for: “Sickenin’ in the rain” – increased risk of gastrointestinal and respiratory infections after urban pluvial flooding in a population-based cross-sectional study in the Netherlands
Source: BMC Infect Dis. 2019 May 2;19:377. doi: 10.1186/s12879-019-3984-5 (PMC6498475; doi:10.1186/s12879-019-3984-5)
Supplement: Supplementary file 2 — Table S2. Results of univariate analyses for ARI in all age categories (overall), children (<16years) and adults. (DOCX 22 kb) [file 12879_2019_3984_MOESM2_ESM.docx]

Table S2: *Results of univariate analyses for ARI in all age categories (overall), children (<16years) and adults (factors with a p-value <0.20 are expressed in bold)*

| Model & Covariates | Overall without ARI | Overall with  ARI | OR | Children without ARI | Children with  ARI | OR | Adults without ARI | Adults with  ARI | OR |
| --- | --- | --- | --- | --- | --- | --- | --- | --- | --- |
|  | N (%) | N (%) | (95% CI) | N (%) | N (%) | (95% CI) | N (%) | N (%) | (95% CI) |
| Type of exposure |  |  |  |  |  |  |  |  |  |
| Skin contact | 406 (68) | 121 (88) | 3.9 (2.0, 7.4) | 54 (64) | 16 (89) | NA | 352 (69) | 105 (88) | 3.9 (1.9, 7.9) |
| Droplets of water in the mouth | 12 (2) | 7 (5) | 1.6 (0.5, 4.9) | 4 (5) | 0 (0) | NA | 8 (2) | 7 (6) | 2.9 (1.0, 8.1) |
| Gulp of water in the mouth | 106 (18) | 33 (24) | 1.7 (1.0, 2.8) | 24 (29) | 2 (11) | NA | 82 (16) | 31 (26) | 1.8 (1.1, 3.0) |
| Head submerged | 3 (1) | 2 (1) | 1.4 (0.5, 4.3) | 0 (0) | 1 (6) | NA | 3 (1) | 1 (1) | 1.1 (0.2, 6.7) |
|  |  |  |  |  |  |  |  |  |  |
| Type of activity |  |  |  |  |  |  |  |  |  |
| Cleaning inside | 253 (26) | 67 (40) | 2.2 (1.5, 3.2) | 9 (6) | 0 (0) | NA | 244 (29) | 67 (47) | 2.2 (1.5, 3.3) |
| Cleaning outside | 159 (16) | 54 (33) | 2.2 (1.5, 3.4) | 5 (3) | 0 (0) | NA | 154 (18) | 54 (38) | 2.4 (1.6, 3.7) |
| Played/run/splashed | 71 (7) | 18 (11) | 0.8 (0.4, 1.8) | 46 (30) | 10 (42) | NA | 25 (3) | 8 (6) | 1.3 (0.4, 4.2) |
| Swum | 1 (0) | 2 (2) | 2.8 (1.0, 8.2) | 1 (1) | 2 (8) | NA | 0 (0) | 0 (0) | NA |
| Used rubber boat | 3 (0) | 4 (2) | 0.9 (0.0, 201) | 1 (1) | 2 (8) | NA | 2 (0) | 2 (1) | 4.2 (0.6, 30.8) |
| Walked | 149 (15) | 49 (30) | 2.4 (1.5, 3.8) | 33 (22) | 7 (29) | NA | 116 (14) | 42 (30) | 2.6 (1.7, 4.1) |
| Cycled | 61 (6) | 25 (15) | 1.9 (1.0, 3.5) | 12 (8) | 3 (13) | NA | 49 (6) | 22 (15) | 2.4 (1.2, 4.4) |
| Driven | 104 (10) | 35 (21) | 1.8 (1.1, 3.0) | 4 (3) | 0 (0) | NA | 100 (12) | 35 (25) | 1.8 (1.1, 3.1) |
| Do not know | 2 (0) | 5 (3) | 11.4 (2.2, 59.7) | 0 (0) | 2 (8) | NA | 2 (0) | 3 (2) | 5.7 (1.3, 25.7) |

Note: OR, odds ratio; CI, confidence interval; NA, not applicable (overall analyses exposure vs ARI was not significant for children, so the separate univariate analyses, multivariate model and the interaction term for children were not created)
