# Supplementary material for: “Sickenin’ in the rain” – increased risk of gastrointestinal and respiratory infections after urban pluvial flooding in a population-based cross-sectional study in the Netherlands
Source: BMC Infect Dis. 2019 May 2;19:377. doi: 10.1186/s12879-019-3984-5 (PMC6498475; doi:10.1186/s12879-019-3984-5)
Supplement: Supplementary file 3 — Figure S1. Flowchart discarded information. (DOCX 47 kb) [file 12879_2019_3984_MOESM3_ESM.docx]

Completed questionnaires

Invited households

n = 3,382

Households

n = 699

Individual participants

n = 1,656

Incomplete information

Households

n = 689

Individual participants

n = 1,635

Contradictory exposure information

Households

n = 582

Individual participants

n = 1,158

Analyses

AGE

ARI

Confounders: hay fever and lung anomalies

Confounders: disorder gastro-intestinal tract, reflux and food allergy

Figure S1. Flowchart discarded information.
