# Supplementary material for: “Sickenin’ in the rain” – increased risk of gastrointestinal and respiratory infections after urban pluvial flooding in a population-based cross-sectional study in the Netherlands
Source: BMC Infect Dis. 2019 May 2;19:377. doi: 10.1186/s12879-019-3984-5 (PMC6498475; doi:10.1186/s12879-019-3984-5)
Supplement: Supplementary file 4 — Table S3. Additional analysis of AGE and ARI with regard to their risk factors with and without individuals having chronic diseases. (DOCX 18 kb) [file 12879_2019_3984_MOESM4_ESM.docx]

Table S3: *Additional analysis of AGE and ARI with regard to their risk factors with and without individuals having chronic diseases. (Factors with a p-value <0.05 are expressed in bold)*

| Model & Covariates | AGE with chronic diseased | AGE without chronic diseased | ARI with chronic diseased | ARI without chronic diseased |
| --- | --- | --- | --- | --- |
|  | aOR | aOR | aOR | aOR |
|  | (95% CI) *^a^* | (95% CI) *^a^* | (95% CI) *^b^* | (95% CI) *^b^* |
| Type of exposure |  |  |  |  |
| Skin contact | 3.9 (1.7-8.9) | 5.0 (1.8-13.5) | 3.4 (1.8-6.5) | 3.2 (1.6-6.1) |
|  |  |  |  |  |
| Type of activity |  |  |  |  |
| Water contact |  |  |  |  |
| No (ref) | Ref | Ref | Ref | Ref |
| Yes, no cleaning | 2.9 (1.3-6.4) | 3.2 (1.4-7.6) | 3.2 (1.7-5.9) | 3.0 (1.5-6.1) |
| Yes, cleaning inside | 3.7 (1.5-9.4) | 3.8 (1.4-10.7) | 3.9 (2.2-7.1) | 3.7 (1.9-7.3) |
| Yes, cleaning outside | 6.8 (2.6-17.9) | 8.2 (3.0-22.9) | 4.3 (2.2-8.4) | 5.1 (2.5-10.5) |
| Yes, cleaning in & out | 8.6 (3.7-20.3) | 8.8 (3.5-22.3) | 5.7 (3.1-10.7) | 4.0 (2.0-8.4) |
| Cycled | 2.2 (1.0-4.9) | 2.7 (1.2-6.4) | NA | NA |
| Do not know | NA | NA | 8.8 (1.5-50.7) | 7.4 (0.6-87.4) |

Note: aOR, adjusted odds ratio; CI, confidence interval; ref, reference category; NA, not applicable

*^a^* Adjusted for age, sex

*^b^* Adjusted for age, sex and summer
